# The development of the Conceptual Construct Validity Appraisal Checklist

**DOI:** 10.4102/ajopa.v5i0.121

**Published:** 2023-11-01

**Authors:** Mario R. Smith, Erica Munnik

**Affiliations:** 1 Department of Psychology, Faculty of Community and Health Sciences, University of the Western Cape, Cape Town, South Africa

**Keywords:** conceptual definition, construct validity, operational definition, scale development, measurement

## Abstract

The validation of instruments is integral to sound psychometric assessment practices. Construct validity, as the apex indicator, is established largely based on data-reduction techniques. This bias overlooks the importance of expert-based content validity and alternate approaches to construct validity. The construct definition, object classification, attribute classification, rater identification, scale formation, and enumeration and reporting (C-OAR-SE) framework provides an alternative to traditional approaches to construct validation. However, the theory lacks articulation into an appraisal instrument that can form an integral part of the methodology used for validation studies. There is a need to focus on the conceptual clarity of constructs and its subsequent impact on operationalisation as a prerequisite to data reduction. This study reports on the development of the Conceptual Construct Validity Appraisal Checklist (CCVAC), based on the C-OAR-SE framework. The CCVAC assesses conceptual construct validity as the logical underpinning of instruments. The CCVAC has three sections, assessing theoretical definitions, operational definitions and scoring, respectively. The measure produces subsection and section scores and a global interpretation matrix. The construction followed a rigorous five-step process. The CCVAC was piloted on the Emotional Social Screening Tool for School Readiness (E3SR). The CCVAC performed well and appears non-sensitive to theoretical frameworks. The E3SR achieved a moderate level of construct validity as estimated by the CCVAC. The kappa statistic (0.55) indicated a moderate inter-rater agreement. All ethics principles were adhered to.

**Contribution:**

The CCVAC is theoretically grounded and provides a quantifiable methodology to objectively assess conceptual construct validity. The CCVAC makes the methodology underpinning construction explicit and produces quantifiable outcomes.

## Introduction

Culture-fair assessment practice is contingent on the availability of reliable and valid measures (Sousa & Rojjanasrirat, 2011). Validation is an important aspect of research and development in test construction. Validation assesses the conceptual definition and the content of the construct being measured (Heale & Twycross, 2015). Face, content and construct validity are typically established (see Laher and Cockcroft (2019) for a comprehensive review of validity).

Traditional approaches to construct validity use factor analysis and data reduction (Yoon et al., 2011). A detailed review of factor analytic approaches is beyond the scope of this paper. Refer to Zygmont and Smith (2014) for a comparison of factor analytic approaches. In factor analytic applications, the fit indices constitute evidence for construct validity (Heale & Twycross, 2015). The correlation between two measures is not sufficient evidence that two instruments are measuring the same construct (Welch, 2011). Furthermore, if two instruments are measuring the same construct, it cannot be assumed that they measure the *right* construct or that their theoretical and operational definitions are comparable (Wiley, 2002). Data reduction is restrictive, as full meanings are not captured completely during analysis (Lakens et al., 2018). Activities of measurement are prioritised over comprehensive theorising of the construct (Lakens et al., 2018). Thus, it is crucial to develop sound theoretical and operational definitions of the construct being measured.

Rossiter (2016) underscored the need to examine the validity of how constructs are defined conceptually (i.e. construct validity). Construct validity is the degree to which performance on a measure represents the level of ability or degree of the construct being measured (Messick, 1988). There is greater value to ensuring that the measure accurately represents the construct as defined (Diamantopoulos, 2005).

Rossiter (2011) recommended the use of qualitative approaches to enrich validation studies. Similarly, Munnik and Smith (2019) reported that non-traditional conceptual frameworks make validation studies more rigorous. Despite these recommendations, the adoption of alternate approaches to establishing validity remains a focus of research. Rossiter (2002) responded by developing the construct definition, object classification, attribute classification, rater identification, scale formation, and enumeration and reporting (C-OAR-SE) framework as a rational rather than an empirically based theory.

The C-OAR-SE method focuses on the development of a highly aligned purpose, the theoretical definition of constructs, empirical item-writing and scoring procedures (Finn & Kayande, 2005). It is both a theory and a procedure that is testable through the evidence of logical argument (Rossiter, 2012). The C-OAR-SE framework was criticised for lacking articulation into an instrument to evaluate the validation process. The development of an appraisal tool to evaluate construction processes and assess validity was needed. This manuscript reports on the development of the Conceptual Construct Validity Appraisal Checklist (CCVAC) as an operationalisation of the C-OAR-SE framework.

### Theoretical framework

The C-OAR-SE model for scale development provides a framework for aligning theoretical definitions and operational activities to produce reliable levels of construct validity (Rossiter, 2012).

#### Construct definition

This step focuses on the definition of the construct under measurement. The definition should be derived from the intended use of the construct and from theory (Rossiter, 2011). The intended use must precede the definition of the construct. The sound and rational definition of the construct being measured contributes to the conceptual construct validity of measures (Rossiter, 2016). This framework prioritises the definition of the construct above the representative measurement (Rossiter, 2011). The researcher is responsible for clearly defining the construct conceptually. Proper definition of the proposed construct is the first part of construct validity. The second part entails the correct classification of the construct in terms of (1) the object to be measured (i.e. object representation), (2) the attribute on which it is measured and (3) the person who is rating the object (Rossiter, 2016). Thus, construct validity relies on sound conceptual definition and not on psychometric testing.

#### Object representation

A conceptual definition of a construct requires the definition of the object that is being measured, indicating constituent components (Rossiter, 2002). Object representation refers to the classification of the principal object as ‘concrete singular’, ‘abstract collective’ or ‘abstract formed’. A *concrete singular* object is unambiguous, with only one meaning, and is described identically by raters (Rossiter, 2011). In social sciences, concrete singular objects do not exist. Psychological constructs may be concrete but are seldom unambiguous. In market research, there are more examples of concrete singular objects. For example, Coke refers to a soft drink packaged in a universal manner and made following a trademarked recipe. This object is universally recognised and identified with absolute consensus. An *abstract collective* object refers to a set of concrete objects that, in the opinion of experts, jointly form a category (Rossiter, 2016). The grouping of the objects is abstract, but the objects are concrete, for example, emotional management, social skills. *Abstract-formed* objects have different components, interpretations and measures (Rossiter, 2011). For example, emotional or social competence has different domains such as sense of self, emotional management and social skills.

#### Attribute classification

An attribute is the dimension being judged. Attributes of the construct being measured are classified as ‘concrete perceptual’, ‘concrete psychological’, ‘abstract achieved’ or ‘abstract dispositional’ (Rossiter, 2011). *Concrete perceptual* attributes are unambiguous to raters and are self-reportable. These attributes have one meaning (concrete) and can consciously be observed (perceptual). For example, to play cooperatively. A *concrete psychological* attribute is inferred by the researcher. For example, a child is able to concentrate if the child can focus on the task at hand. An *abstract achieved* attribute has multiple components that comprise the attribute and is formed or achieved, such as knowledge (Rossiter, 2011). *Abstract dispositional* attributes are inferred by the researcher and are not directly perceived by the rater such as emotion regulation or self-esteem:

#### Rater-entity identification

The rater-entity identification refers to the decision about who will conduct ratings. The rater perspective is the final consideration in the conceptual definition of a construct (Rossiter, 2002). *Expert* raters are trained professionals, while *individuals* are the raters in self-report measures. A *group* rater entity is a representative of a particular group (Rossiter, 2011).

#### Selection of item-type and answer scale

In this step, decisions must be made about the item format and response options. Items must be based on the alignment between object representation and attribute classifications (Rossiter, 2011). Pre-testing establishes whether items are understood as intended (Rossiter, 2011).

#### Enumeration and scoring rules

The enumeration and scoring rules pertain to how the scoring is derived. Scoring rules also relate to the manner in which scores are combined to create group statistics (Rossiter, 2011). Scoring rules dictate the scale totals and subsequent interpretations.

The C-OAR-SE framework yields adequate confirmation that the definitions of constructs for different instruments are compatible when establishing convergent validity (Rossiter, 2012). It addresses the bias towards data reduction as a sufficient indicator of construct validity. The C-OAR-SE framework informed the aim of the study, the methodology employed and constituted the theoretical underpinning of the resultant appraisal checklist.

## Methods

### Aim of the study

The study aimed to develop a measure to assess the conceptual construct validity underpinned by the C-OAR-SE framework.

### Design

This two-phased construction study included: (1) a construction phase and (2) a pilot study.

#### Phase one

The construction followed five steps:

- developing a theoretical structure for the instrument,
- the format of the instrument and scoring guidelines were decided upon,
- a pool of items was generated and a draft instrument developed,
- the draft scale was reviewed and refined and
- accompanying documents were prepared.

**Theoretical structure:** The C-OAR-SE framework formed the theoretical underpinning of the proposed measure. The measure was intended to define the C-OAR-SE for measurement (i.e. operationalisation). The resultant measure is called the Conceptual Construct Validity Appraisal Checklist, abbreviated as the CCVAC (Appendix 1). The CCVAC assesses whether a measure has construct validity based on the process of conceptualising the construct to be measured. It is intended to be used when selecting an instrument or developing new measures.

**Format of the instrument:** The checklist format was selected. Each item corresponded to criteria in the C-OAR-SE formulation. The checklist was divided into three sections. Section 1 dealt with the theoretical definition of the construct. It assessed whether the constituents and components of the construct were defined properly relative to the intended purpose of the measure. Section 2 dealt with deconstruction or operationalisation of the construct for measurement. The section evaluated the process followed to operationalise the construct. This section consisted of three subsections. The subsections assumed that good practice includes a logic model in which theoretical definitions are articulated into measurements. Subsection 1 addressed the nature of the construct being measured; subsection 2 assessed the nature of the attributes being measured and subsection 3 evaluated the technical aspects of the scale. This section includes two subsections that deal with (1) scale formation and (2) enumeration.

A sliding scale was adopted where higher scores indicated a higher quality response. Each subsection generated a score based on summed items. Subsection scores were summed to derive section scores. Scoring was conceptualised as a cumulative process, with scores interpreted independently for sections and cumulatively for global scores.

A quality description indicated the extent to which section outcomes had been achieved. The section scores were triangulated to derive a global outcome in an interpretation matrix. The global score had corresponding corrective actions.

**Item generation:** Items across sections evaluated the logic underlying the conceptualisation of the construct. The items addressed the essence of the C-OAR-SE criteria and did not use the technical language of Rossiter’s formulation. This reduced bias related to the theoretical assumptions, language and process implications from the C-OAR-SE. The draft checklist consisted of 35 items comprising 5, 18 and 12 items in Section 1, Section 2 and Section 3, respectively.

**Refining the scale:** The draft checklist was reviewed in two phases by four reviewers with expertise in research and test construction, as evidenced by their academic qualifications, work history and research outputs. In the initial review, two clinical psychologists, registered with the Health Professions Council of South Africa (HPCSA), found that the aim of the measure was clear. However, they identified the risk that the measure could only be used by those who were familiar with the C-OAR-SE formulation, as the items were aligned with the language of the framework. The draft was substantially revised to achieve a greater level of neutrality by focusing on the process rather than technical formulations.

The revised draft had 27 items with five items in Section 1, 11 items in Section 2 and 11 items in Section 3. Subsequently, two research psychologists, registered with the HPCSA, identified that the items on attributes were difficult to score as they were not familiar with the theoretical references. The wording of the items was revised to be more generic and less reflective of a specific theoretical position.

The scoring was finalised as follows. Section 1 produces a maximum score of 7. Section 2 produces a maximum score of 16, including the scores for Subsection 1 (maximum of 4), Subsection 2 (maximum of 6) and Subsection 3 (maximum of 6). Section 3 produces a maximum score of 13, comprised the scores for Subsection 1 (maximum of 6) and Subsection 2 (maximum of 7).

**Interpretation:** Three outcomes were used to describe section scores, namely: (1) not achieved, (2) partially achieved and (3) achieved. Section 1 describes whether conceptual definitions have been achieved. Section 2 describes whether correct classification has been achieved. Section 3 described whether sound technical and scalar decisions have been taken.

An interpretation matrix was designed for the global outcome that indicates whether construct validity was achieved. Each section score is plotted into the matrix. Three possible global outcomes were identified, with corresponding corrective actions. High construct validity is achieved when all section scores are categorised as ‘achieved.’ A poor level of construct validity is reflected when all section scores are categorised as ‘not achieved.’ When section scores reflect a mixture of full or partial achievement, a medium level of construct validity is achieved. Two possible outcomes are possible for a medium level of construct validity. Firstly, a low medium level of construct validity will be achieved when all three sections were scored as partially achieved or if one section had full achievement while the other two remained partially achieved. Secondly, a high medium level of construct validity will be denoted by two sections that are described as fully achieved and one that was partially achieved.

A high or medium level of construct validity is considered appropriate to proceed with establishing the psychometric properties. Users may decide to use high medium only if they want to see a more stringent threshold.

**Developing accompanying templates:** The CCVAC template is completed by the researcher(s) responsible for the construction or selection of the measurement (Appendix 2). This template corresponds to the sections of the checklist. The completed template is used by reviewers to evaluate the conceptual clarity of the measure under investigation. The template provides uniformity in the presentation of required information on the measure being evaluated. It reduces bias against researchers who are unfamiliar with the C-OAR-SE by prompting for the required information.

#### Phase two

Piloting entailed an application of the CCVAC to the Emotional Social Screening Tool for School Readiness (E3SR). The E3SR is a South African screening instrument that measures social-emotional competence in preschool children. The six factors include emotional maturity, emotional management, sense of self, readiness to learn, social skills and communication. Munnik et al. (2021) reported good psychometric properties for the E3SR. The C-OAR-SE was not used in the construction of the E3SR that assisted in testing out the potential impact of familiarity with the framework.

Two independent reviewers participated in the piloting. Reviewer 1 (R1) was a research psychologist with expertise in test construction. Reviewer 2 (R2) was a researcher with experience in questionnaire design and statistical analysis.

### Instrument

The CCVAC was used to appraise construct validity.

### Procedure and data analysis

The developer of the E3SR recorded the details of the construction processes of the E3SR on the CCVAC template. The completed template was used for the evaluation. Inter-rater reliability was computed using the kappa statistic as recommended by Glen (2014).

### Ethical considerations

The Humanities and Social Science Research Ethics Committee (HSSREC) gave ethics clearance (Ref no: HS19/2/4). Permission was given by Dr Munnik to use the E3SR for piloting of the CCVAC. All personal data of reviewers were de-identified and stored in line with the specified guidelines of the *Protection of Personal Information Act* (*POPIA*). Raters signed a binding agreement to uphold copyright and intellectual property stipulations of the E3SR and the CCVAC to maintain independence of their contributions.

## Results

The three sections of the CCVAC represent discrete conceptual parts of the C-OAR-SE. Items mirror the essence of the different sections. Feedback on the first draft identified that the alignment of the structure of the CCVAC and template with the C-OAR-SE framework was useful. The reviewers felt that the items and template used technical terms from the framework, which posed a challenge. Firstly, it was biased towards construction done in the framework. Secondly, if constructors were not familiar with the technical language of the framework, they would provide limited information that in turn would impact appraisal adversely. Consequently, the draft checklist was substantially revised to ensure neutral language and wider application that was not contingent on the framework. Items and prompts were rewritten in general construction language.

The reviewers reported that the CCVAC template was crucial. It provided the information in an accessible manner and made evaluation and scoring easier. However, the scoring process was initially confusing. The reviewers recommended separating the scoring and interpretation guide from the checklist. The scoring was made clearer and presented after the sections.

The kappa statistic (0.55) tested significant at a 0.00 alpha level. There was a moderate agreement between the raters on the extent to which the E3SR achieved construct validity. The reviewers assigned identical scores on Sections 1 and 3. Inter-rater reliability was negatively impacted by disagreement on one subsection in Section 2, as illustrated in Table 1. Reviewer 1 reported not fully understanding the items and attributed lower scores ostensibly because of unfamiliarity with the C-OAR-SE.

**TABLE 1:** Comparison of reviewer scores.

Item	Reviewer 1	Reviewer 2
**The nature of the attributes being measured**		
Is there evidence that the nature of the attributes was considered in the process of operationalisation?	1	2
Does the stated nature of the attributes align with the theoretical definition?	1	2
Does the inferred classification align with the theoretical definitions?	1	2
**Subsection score**	**3**	**6**

## Discussion

The findings suggest that the E3SR achieved a moderate level of construct validity, as estimated by the CCVAC. There was a significant agreement between raters at a moderate level (kappa statistic of 0.55, *p* = 0.00). The inter-rater reliability was negatively impacted by disagreement on the attribute subsection. The items require familiarity with the notion of classification, as articulated in the C-OAR-SE. The C-OAR-SE guidelines are established but not well known. The addition of instructions or explanations may improve review quality and increase inter-rater agreement. It was important to ensure that the CCVAC informed the process evaluation and that the language was not overly reflective of the theory. The CCVAC successfully operationalised the C-OAR-SE guidelines for establishing construct validity. The CCVAC addresses the lack of a formal checklist to evaluate conceptual construct validity. The reduced agreement may reflect the lack of attention to conceptualisation rather than a lack of familiarity with the C-OAR-SE formulation.

### Limitations

The CCVAC was piloted on only one instrument. The findings, although encouraging, need to be replicated on more instruments. The CCVAC must be interpreted relative to the C-OAR-SE, as criteria contained in other guidelines may not be accommodated equally. The language was adapted to reduce dependence on familiarity with the framework. Similarly, the use of a template for recording the source information reduces bias and ensures that the required information is captured before scoring. The impact of the framework will remain a focus of further refinement and research.

## Conclusion

The CCVAC is theoretically grounded and provides a quantifiable methodology to objectively assess conceptual construct validity. The CCVAC appears to be a robust measure of construct validity, which is not sensitive to theoretical frameworks.

### Implications for future research, practice and theory

The CCVAC is an operationalisation of the C-OAR-SE. The format allows developers to assess their process of test construction before piloting. It offers a thorough process and quantifiable appraisal against criteria, regardless of the theoretical framework espoused, in the construction process. The CCVAC centralises construct definition and constitutes a means for evaluating the construction process empirically from a theory-driven perspective. The checklist makes the methodology underpinning construction explicit and produces quantifiable outcomes.

## Appendix 1

### The Conceptual Construct Validity Appraisal Checklist (CCVAC)

#### Authors: M. Smith and E. Munnik

***Intended user group:*** The CCVAC is designed to be used by trained professionals with a working knowledge of test construction, instrument development and measurement.

This checklist is based on the C-OAR-SE framework proposed by Rossiter (2011). The CCVAC attempts to assess whether the construct being measured has been defined properly. This constitutes a qualitative process of achieving construct validity at a theoretical or conceptual level. Well-defined constructs produce coherent instruments that can then be used judiciously to test construct validity using data-reduction techniques.

The CCVAC consists of three subsections that are aligned with the C-OAR-SE.

***Section 1:*** Theoretical definition

***Section 2:*** Operational classification

***Section 3:*** Technical aspects

**Table:**

***Section 1: Theoretical (construct) definition***			
This section assesses whether the construct has been defined properly at a conceptual or theoretical level in terms of its constituents and components.			

**Criterion**	**Guide**	**Scores**	

Was the intended use (purpose) of the construct clarified?		Yes	1
		No	0
Was a theoretical definition provided?		Yes	1
		No	0
Was the definition rational?		Yes	2
		Partially	1
		No	0
Was the definition clear and unambiguous?		Yes	2
		Partially	1
		No	0
Did the intended use precede the definition of the construct?		Yes	1
		No	0
**Section score /7**			

***Section 2: Operational definitions (construct classification)***			
This section assesses the process by which the theoretical definition was deconstructed or operationalised for measurement. The aim is to evaluate the process followed to operationalise (classify) the construct.			

(i) The nature of the construct being measured *(object classification)*			
Was there a classification process?	Yes – classification was explicit	Yes	2
	Partially – classification was implicit	Partially	1
	No – no classification was attempted	No 0
What kind of classification does the construct approximate?	*Concrete singular*: The construct has a singular meaning that is understood universally.		
	*Abstract collective*: The construct is comprised of multiple components that form a single meaning entity or unit.		
	*Abstract formed*: The construct has multiple possible meanings.		
Does the inferred classification align with the theoretical definition(s)?		Yes	2
		Partially	1
		No	0
**Subsection score /4**			
(ii) The nature of the *attributes being measured*			
Is there evidence that the nature of the attributes was considered in the process of operationalisation?		Yes	2
		Partially	1
		No	0
Does the stated nature of the attributes align with the theoretical definition?		Yes	2
		Partially	1
		No	0
What classification does it approximate?	*Concrete perceptual*: A self-reportable attribute, within the conscious awareness of the person, that has only one meaning.		
	*Concrete psychological*: An attribute that is not within the conscious awareness of the person and cannot be self-reported. It must be inferred by an observer or rater.		
	*Abstract achieved*: An attribute with multiple components that are outlined clearly in conceptual definition. The attribute is something that is formed or achieved (e.g. knowledge) and can be perceived directly by the rater.		
	*Abstract dispositional*: An attribute that has multiple components that are clearly outlined in the conceptual definition. The attribute cannot be perceived directly by the rater and must be inferred by the researcher or test developer.		
Does the inferred classification align with the theoretical definitions?		Yes	2
		Partially	1
		No	0
**Subsection score /6**			
(iii) *Rater identification*			
Have specific raters been identified?	Raters are the individuals who will complete the instrument or respond directly to the items.	Yes	1
		No	0
Was a category of raters selected?	Individuals: The *individual* is considered the rater in self-report measures. Groups: A *group* rater-entity is considered a representative sample of a group. Experts: Trained professionals or experts who will perform or conduct ratings.	Yes	1
		No	0
Was the selected rater aligned with the theoretical definition?		Yes	1
		No	0
Was the selected rater aligned with the intended use of the instrument?		Yes	1
		No	0
Was the rater aligned with the operational definition?		Yes	1
		No	0
Was the rater aligned with the nature of the attributes?		Yes	1
		No	0
**Subsection score /6**			

**Section 3: Technical components**			
This section assesses the technical components of the scale including: (1) scale formation and (2) enumeration and reporting.			

(i) *Scale formation*			
Has an item-type been selected?		Yes	1
		No	0
Has an answer scale/response option been selected?		Yes	1
		No	0
Was the item format pre-tested?		Yes	1
		No	0
Were the items understood as intended by the raters?		Yes	1
		No	0
Was there a process reported whereby the relationship (alignment) between scale items, and the nature of the construct and attributes was considered.		Yes	2
		Partially	1
		No	0
**Subsection score /6**			
(ii) *Quantification and reporting*			
Have scoring rules been developed?		Yes	1
		No	0
How were scoring rules derived?		From single items	1
		Across items	2
Were rules developed for combining individual and group scores?		Yes	1
		No	0
Were the scoring rules developed for interpreting the scale totals?		Yes	1
		No	0
Can scale scores be interpreted as an indicator of the construct being measured?		Yes	1
		No	0
If group statistics can be derived, were any rules developed to create group statistics?		Yes	1
		No	0
**Subsection score /7**			

### Scoring Guide

The CCVAC is scored on three levels.

#### Items

The CCVAC includes individual and composite items. The individual items on the CCVAC are scored as follows:

- Yes = 1; No = 2.
- Where indicated, composite items generate scores of 3, 2, and 1 respectively.

#### Subsection scores

Subsection scores are generated by the summation of individual items in that subsection.

#### Section scores

The CCVAC produces a score for each section that is derived across items and subsections, where applicable. The score is an indication of the extent to which the objective of that section has been achieved.

Scores are allocated as follows:

3 – Achieved

2 – Partially achieved

1 – Not Achieved

**Interpretation guide: Section 1**

**Table:**

Was the proposed construct properly defined?		
Yes	Affirmative answers must be derived on all five questions above. Maximum score obtained for this subsection would be 7.	3
Partially	The first two questions have negative answers. Scores obtained range between 3 and 6.	2
No	Fewer than three affirmative responses recorded above. Score for this subsection would be < 3.	1

**Interpretation guide: Section 2**

**Table:**

Was the proposed construct adequately classified?		
Yes	Maximum score achieved on all three subsections.	3
Partially One of the three may apply	Maximum score obtained on at least one subsection and three affirmative responses on the remaining two subsections. At least four affirmative responses in two subsections and three affirmative responses in the remaining subsection. At least three affirmative responses on all three subsections.	2
No	Fewer than three affirmative responses across subsections.	1

**Interpretation guide: Section 3**

**Table:**

Were the technical components adequately addressed?		
Yes	Maximum score achieved on both subsections.	3
Partially One of the two may apply	Maximum score obtained on at least one subsection and three affirmative responses on the remaining subsections. At least three affirmative responses on both subsections.	2
No	Fewer than three affirmative responses across subsections.	1

**Global outcome: Enter the section score on the table below**

**Table:**

Subsection	Rating		
	Yes (Fully achieved)	Partially	No
1	Sound conceptual definition of the construct	Partially correct conceptual definition of the construct	Poor conceptual definition of the construct
2	Sound and correct classification	Partially correct classification	Poor classification
3	Sound technical scalar decisions	Partially sound technical scalar decisions	Poor technical scalar decisions
**Construct validity**	**HIGH**	**Low MEDIUM** All three sections are partially achieved One section was fully achieved & 2 sections partially achieved **High MEDIUM** Two sections were fully achieved & 1 partially achieved	**POOR**
**Action**	***Proceed with psychometric testing***	***Cautiously proceed with psychometric testing***	***Revise instrument and repeat conceptual assessment***

## Appendix 2

### Conceptual Construct Validity Appraisal Checklist Template

**Table:**

**1. Construct definition** This section assesses whether the construct has been defined properly at a conceptual level in terms of its constituents and components.		
**Criterion**	**Description**	**Response**
Conceptual definition	Describe how the construct was derived. In your description comment on the extent to which the theoretical and intended use of the construct was considered.	
	Indicate the temporal order of the intended use and the definition of the construct. In other words, which came first?	
	Provide the theoretical definition of the construct(s) under evaluation.	
	Provide a motivation or rationale for the definition you adopted/developed.	
**2. Construct classification** This section assesses whether the construct has been correctly classified. Three aspects are assessed.		
*1. The object to be rated*	Describe the process followed to classify the object.	
	Indicate which of the following best described the classification of the object: Not Applicable Concrete singular Abstract collective Abstract formed	
	Motivate your classification as indicated above. Make reference to the alignment of the object classification with the definition.	
*2. Attribute classification*	Describe the process followed to classify the attributes of the construct.	
	Which of the following best reflects the categorisation of the attributes of the construct? Not applicable Concrete perceptual Concrete psychological Abstract achieved Abstract dispositional	
*3. Rater classification*	Identify who is eligible to rate the construct being measured.	
	Which category best describes the classification of raters? Not applicable Individuals Groups Experts	
	Motivate your response above.	
	Describe the alignment of the rater classification with the theoretical definition.	
	Describe the alignment of the rater classification with the object classification.	
	Describe the alignment of the rater classification with the attribute classification.	
	Describe the alignment of the rater classification with the intended use of the instrument.	
**3. Technical component** This section assesses the technical components of the scale. Two aspects are assessed, namely: (1) scale formation and (2) enumeration and reporting.		
*1. Scale formation*	Describe the item type that was selected.	
	Describe the answer scale that was selected.	
	Describe how the production of the scale items was informed by the alignment between the definition, object representation, and attribute representation.	
	Describe any processes aimed at checking whether the items were understood as intended by the raters.	
*2. Enumeration and scoring*	Describe the scoring rules across items.	
	How were the scoring rules derived? In your answer, indicate whether scoring rules were from single items and/or across items.	
	Do the scoring rules make group statistics possible?	
	Do the scoring rules make provision for the combination of individual scores and group scores? Describe the process.	
	Describe how scale totals are interpreted using the scoring rules.	
	Describe how scale scores are interpreted as an indicator of the construct being measured.	
